# Habitual use of GPS negatively impacts spatial memory during self-guided navigation

**DOI:** 10.1038/s41598-020-62877-0

**Published:** 2020-04-14

**Authors:** Louisa Dahmani, Véronique D. Bohbot

**Affiliations:** 1 Integrated Program in Neuroscience, McGill University, Montreal, Quebec H3A 2B4 Canada; 2 Douglas Mental Health University Institute, Department of Psychiatry, McGill University, Montreal, Quebec H4H 1R3 Canada

**Keywords:** Spatial memory, Human behaviour

## Abstract

Global Positioning System (GPS) navigation devices and applications have become ubiquitous over the last decade. However, it is unclear whether using GPS affects our own internal navigation system, or spatial memory, which critically relies on the hippocampus. We assessed the lifetime GPS experience of 50 regular drivers as well as various facets of spatial memory, including spatial memory strategy use, cognitive mapping, and landmark encoding using virtual navigation tasks. We first present cross-sectional results that show that people with greater lifetime GPS experience have worse spatial memory during self-guided navigation, i.e. when they are required to navigate without GPS. In a follow-up session, 13 participants were retested three years after initial testing. Although the longitudinal sample was small, we observed an important effect of GPS use over time, whereby greater GPS use since initial testing was associated with a steeper decline in hippocampal-dependent spatial memory. Importantly, we found that those who used GPS more did not do so because they felt they had a poor sense of direction, suggesting that extensive GPS use led to a decline in spatial memory rather than the other way around. These findings are significant in the context of society’s increasing reliance on GPS.

## Introduction

When we navigate in a new environment, we are required to pay attention to our surroundings and to update our position using our own internal navigation system in order to reach our destination. Using GPS removes these requirements and renders navigation less cognitively demanding. In fact, people who travel along given routes using GPS gain less knowledge about those routes compared to people who travel the same routes without an aid, using a map, or after being guided by an experimenter^1–5^. However, no studies have looked at whether GPS use has long-term effects on our internal navigation system, when we are required to find our way without a navigation aid.

When we navigate without GPS in a new environment, there are two navigation strategies that we can use that depend on separate brain systems. One is the spatial memory strategy and involves learning the relative positions of landmarks and serves to form a cognitive map of the environment^6,7^. This strategy critically relies on the hippocampus^6,8–11^, a brain region heavily involved in episodic memory^12^ and relational memory^13^. The other strategy is the stimulus-response strategy and involves learning a sequence of motor responses (e.g., turn left) from specific positions (e.g., next corner). Stimulus-response learning critically relies on the caudate nucleus^14,15^, a brain region also responsible for habit learning (e.g., learning how to ride a bicycle)^16,17^. This strategy leads to more rigid behavior and allows us to navigate on ‘auto-pilot’ on routes that we travel frequently. Our tasks allow us to measure several facets of navigation, including the extent of navigation strategy use (people can use the same strategy but rely on it to different extents), learning (how quickly people learn about a new environment), cognitive mapping, landmark encoding and reliance, and flexibility/rigidity. The spatial memory and stimulus-response strategies are distinct as they rely on separate neural networks and demonstrate a double dissociation, in that lesioning the spatial memory neural circuit impairs spatial memory but spares stimulus-response learning, while lesioning the stimulus-response neural circuit impairs stimulus-response learning but spares spatial memory^11,15,18–25^. Thus, navigation is a broad process that includes two distinct methods: spatial learning and memory and stimulus-response learning and memory.

Using GPS involves following step-by-step sensorimotor instructions, which is similar to learning stimulus-response associations (e.g., turn right at the next intersection, turn left in 500 m). In a cross-sectional study, we sought to determine whether individuals with greater GPS habits rely more on stimulus-response strategies and less on spatial memory strategies when they are required to navigate without GPS, and whether they have poorer cognitive mapping abilities and landmark encoding. We then performed a three-year follow-up in which we retested a small subset of participants. This longitudinal session served to investigate whether GPS use has a negative impact on the various spatial memory facets over time.

## Methods

### Participants

Sixty healthy young adults between the ages of 19 and 35 participated in the cross-sectional study. Participants were required to be right-handed and to have no history of neurological or psychiatric disorders, or of alcohol or drug abuse. Additionally, they had to have no history of head trauma followed by a loss of consciousness. At the time of recruitment, participants had to be local regular drivers, defined as driving at least 4 days a week in Montreal, Canada. We had no requirements for GPS use, as the majority of participants used GPS at least once a week. To reduce the potential influence of outliers, we excluded individuals with values three standard deviations away from the mean. We chose this conservative threshold as our sample was comprised of healthy young adults. One outlier was found in terms of lifetime GPS experience. No outliers were found in terms of the other GPS habits we measured, i.e. hours of GPS use since pre-test, sense of GPS dependence, or GPS reliance (see Questionnaires section). Of the 60 individuals who participated, ten were excluded: six were not regular drivers, two were not motivated and did not learn or complete the tasks, one was ambidextrous, and one was an outlier in terms of lifetime GPS experience. Fifty participants were included in the cross-sectional study (18 women, 32 men; mean age: 27.6 ± 4.5 years old; see Table 1 for demographic information). All 50 participants were invited for a long-term follow-up session. This session was unplanned and therefore participants had not initially agreed to return for a follow-up. Given that the sample was primarily undergraduate students, many participants were unreachable or had moved away from the city three years after initial testing, and therefore a small subset of 13 participants (4 women, 9 men; mean age: 28.46 ± 3.93 years old; Table 1) came back for a follow-up assessment (mean delay between pre- and post-testing = 3.23 ± 0.50 years). Men and women did not differ on any of the GPS measures. One participant in the cross-sectional study did not do the CSDLT due to lack of time. At baseline, the longitudinal sample did not differ from the remaining participants in the cross-sectional sample in terms of demographics, GPS variables, SBSOD, or navigation variables (independent samples t-tests: all Bootstrap BCa 95% CI crossed 0; Chi-square test for difference in proportion of men and women: *p* > 0.05). The Institutional Review Board of the Douglas Mental Health University Institute approved the study and all research was performed in accordance with its guidelines and regulations. Informed consent was obtained from all participants.

**Table 1:** Participant demographics and average neuropsychological test scores at initial testing.

	Women/Men	Age (years)	Education (years)	TONI-3 IQ*	RAVLT	ROCF*
Cross-sectional sample (N = 50)	18/32	27.6 (4.5)	16.6 (2.7)	104.9 (17.0)	11.1 (2.8)	21.8 (7.6)
Longitudinal subsample (n = 13)	4/9	28.5 (3.9)	17.1 (2.9)	108.8 (16.4)	11.9 (2.8)	21.8 (8.0)

Standard deviations are shown in parentheses.

^*^Information is missing for one participant.

TONI-3: Test of non-verbal intelligence-3 (quotient); RAVLT: Rey Auditory Verbal Learning Test (delayed recall); ROCF: Rey-Osterrieth Complex Figure (delayed recall); SBSOD: Santa Barbara Sense of Direction scale.

### Virtual navigation tasks

We used two virtual navigation tasks in this study. They were developed using Unreal Tournament 2003 development kit (Epic Games, Raleigh, NC).

### Concurrent spatial discrimination learning task (CSDLT)

**Figure 1:**
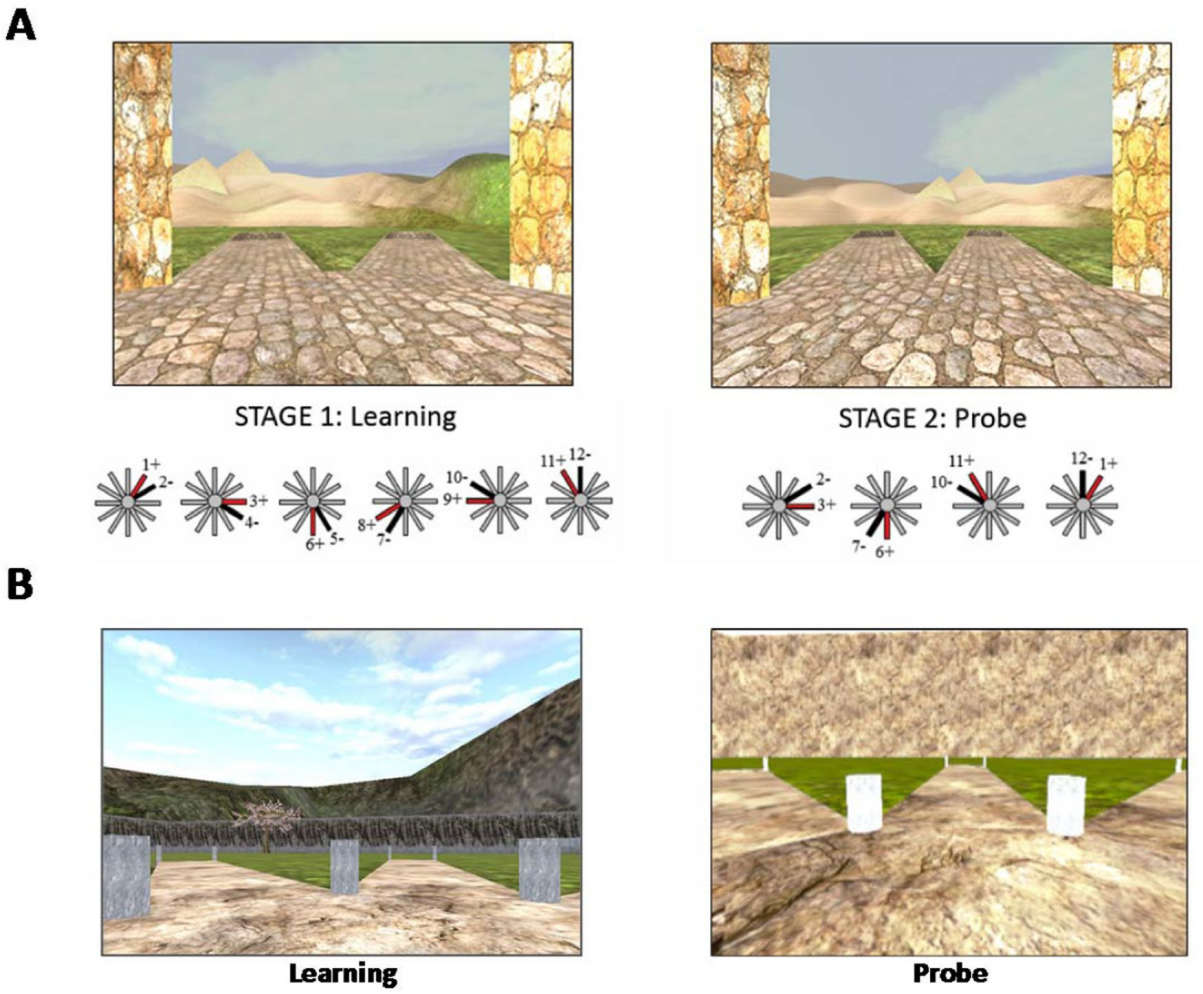
Virtual navigation tasks. (**A**) The Concurrent Spatial Discrimination Learning Task (CSDLT) consists in a 12-arm radial maze surrounded by landmarks. The paths are divided into six pairs of paths. In the learning stage (left), participants are presented with each pair and have to learn which paths contain an object, by using either a spatial memory strategy or a stimulus-response strategy. Once participants learn the task to criterion, they are taken to the probe stage (right), where the paths are recombined into new pairs of adjacent paths. However, the objects remain in the same paths. Those who used a spatial memory strategy during learning make fewer errors, as they learned the precise spatial relationship between the target arms and the landmarks. Those who used a stimulus-response strategy make more errors, as the motor action that they learned will lead them to the incorrect path when presented with the learned stimulus. (**B**) The 4-on-8 Virtual Maze (4/8 VM) consists in an 8-arm radial maze surrounded by landmarks. In Part 1, four of the paths are blocked and four are open. Participants have to retrieve objects at the end of the open paths. In Part 2, the barriers are removed. Participants have to avoid the paths they visited in Part 1 to retrieve the remaining objects. They can learn the object locations using a spatial memory strategy or a stimulus-response strategy. Once participants learn the task to criterion, they are taken to a probe stage, where a wall is raised around the maze that hides the landmarks. People who used a spatial memory strategy during learning make more errors than people who used stimulus-response strategies, as they can no longer use landmarks to find the target paths.

The CSDLT is a virtual human analogue of a task developed for mice^26^. The task consists of a 12-arm radial maze that is surrounded by a rich landscape and environmental landmarks, such as a mountain range, trees, pyramids, a pond, etcetera (Fig. 1A). This task has been shown to be sensitive to fMRI BOLD activity and grey matter in the hippocampus^27–30^. There are two stages in the task; a learning stage (Stage 1) and a probe stage (Stage 2).

Stage 1: The 12 paths diverge around a central platform. At the end of each of the 12 paths, there is a staircase that leads down to a pit, where an object can be found in some of the paths. The paths are divided into six pairs of adjacent paths that are presented separately. In a trial, participants start at the central platform and face each pair of paths, one after the other, in a pseudo-random order. When they are faced with a pair of paths, they have to go down one of the paths. Only one of these contains an object. Participants can learn the location of the objects by using either a hippocampus-dependent spatial memory strategy, where they learn the precise spatial relationship between the target path and a landmark/feature in the background (e.g., “The target path is a bit to the right of the pyramids”), or a stimulus-response strategy, where they learn a motor action (e.g., “I have to go left”) in response to a stimulus (e.g., “when I see the pyramids”). Participants visit the various pairs of paths until they reach a learning criterion of 11/12 correct choices within two consecutive trials. Although participants can reach the learning criterion early on, we administer a minimum of six trials for all participants.

Stage 2: Once participants learn the location of the objects, they are given two probe trials. Here, the paths are recombined into new pairs of adjacent paths, such that the perspective is shifted in comparison to the learning trials. For example, path #3, previously presented with path #4 (Fig. 1A, left), is now presented with path #2 (Fig. 1A, right). Only four recombined pairs of paths are shown in each probe trial, as the recombination must be done such that the new adjacent paths only contain one object. The objects remain in the same paths as before. The maximum score for each probe trial is four. Those who used a spatial memory strategy to learn the location of the objects will make fewer errors, as they learned the precise spatial relationship between the target paths and the landmarks. On the other hand, people who used a stimulus-response strategy during learning will make more errors, as the motor action that they learned will lead them to the incorrect path when presented with the learned stimuli (see example in Fig. 1A). Therefore, those who make few errors on the probe trials (performance on probe 1 and 2 combined) are considered to have used a spatial memory strategy during learning. We also investigated performance on individual probe trials as they capture different effects: both trials test the level of spatial memory strategy use, however the second probe trial additionally tests flexibility in learning from one’s mistakes in probe 1. People generally learn from their mistakes in the first probe trial and do better on the second probe trial, as evidenced by a significantly higher probe 2 score compared to probe 1 (paired *t*-test: *t*_(48)_ = 4.16, mean difference = 0.73, Bootstrap BCa 95% CI [0.40, 1.10]). Thus, not improving one’s performance on the second probe trial indicates rigidity, a hallmark of stimulus-response learning.

The dependent variables were the following: Number of trials to criterion and probe score (on the first trial, second trial, and both trials combined).

### 4-on-8 Virtual Maze (4/8 VM)

This task was modeled after a maze task used in rodents^31^. Similar to the CSDLT, it is composed of a radial maze that is surrounded by a landscape and landmarks, such as mountains, trees, and boulders (Fig. 1B, left). This task is sensitive to fMRI BOLD activity and grey matter in the hippocampus^32,33^. The radial maze is made up of eight paths that diverge around a central platform. At the end of each path, a staircase leads down to a pit. Each trial has two parts:

Part 1: Four of the eight paths are blocked by barriers, while the other four are accessible. Participants have to visit the four accessible paths and retrieve an object located in the pit at the end of these paths. They are instructed to memorize the location of the paths containing the objects. Once they have done so, they are taken to Part 2.

Part 2: All the paths become accessible and participants have to visit the remaining arms in order to retrieve the objects. Thus, they have to avoid the paths that they visited in Part 1. Participants can solve this task by either using a spatial memory strategy, where they learn the spatial relationship between the target path and a landmark (e.g., “the objects are located in the paths to the left of the large boulder and to the right of the tree”), or a stimulus-response strategy, where they learn a series of motor actions in response to a stimulus (e.g., “from the starting position, I have to take the path straight ahead and then skip a path on the right”).

Participants had to learn the task to criterion; they had to find the objects in Part 2 without making errors in at least one trial. We administered a minimum of three trials, regardless of when participants reached the learning criterion. Afterwards, we administer a probe trial, where Part 1 is the same as in the learning trials. In Part 2, a wall is raised around the maze, blocking participants’ view of the landmarks (Fig. 1B, right). Here, people who used a spatial memory strategy during learning will make more errors, as they can no longer use the landmarks to find the target paths. The probe is therefore a measure of landmark reliance. At the end of the task, we ask participants to draw a map of the maze to assess their cognitive map of the environment^34^. Here, we assigned one point for every landmark drawn and one point for each landmark that was correctly placed. Then, we administer a verbal report, where we ask participants to describe how they solved the task throughout the trials. The verbal report serves to determine the navigation strategies that participants used. The experimenter administered a structured interview, starting with more general questions (“What did you do to learn which paths to take and which ones to avoid?”, “Can you walk me through an example of what was going through your head as you were doing the task? Can you give me a concrete example?”), and asking more specific questions depending on the participant’s answers. For example, if a participant mentioned using a sequence, the administer would ask “Can you be more specific about how you [counted] the arms? Where did your [sequence] start?”. Experimenters only used terms (e.g., counting, sequence, landmark, point of reference, etc.) that were first mentioned by the participant. Participants were also asked if they used the same strategy throughout the experiment, and whether the starting position in the trials changed or remained the same. Previously, we found spontaneous strategies, i.e. the strategy used in the very first trial, to be associated with increased fMRI BOLD activity and grey matter in our regions of interest. Using a spatial memory strategy was associated with increased fMRI BOLD activity and grey matter in the hippocampus while using a stimulus-response strategy was associated with increased fMRI BOLD activity and grey matter in the caudate nucleus^32,33^. Thus, in the present study, we categorized participants according to their spontaneous navigation strategy. A strategy score is attributed in the following way: 0 points are given if participants used a stimulus-response strategy that did not rely on any landmarks; one point is given when a stimulus-response strategy was used and which relied on one landmark, and two points are given when a stimulus-response strategy was used and where two or more landmarks were used to confirm the paths but were not integral to performance (i.e., participants mainly relied on a full sequence from a single start position); three points are given if a spatial memory strategy was used where several landmarks were relied upon. Two raters assessed the verbal reports to determine the strategies used, and a third rater was used when there was disagreement. The inter-rater reliability was 98% in the cross-sectional study and 100% in the longitudinal study. The verbal report also served to determine the number of landmarks participants used (e.g., “I used the rock and the tree to find the objects”) and the number of landmarks they noticed (e.g., “I saw a mountain, but I did not use it”).

The dependent variables were the following: Number of trials to criterion, average number of errors on Part 2 of the learning trials, navigation strategy score, probe errors, map drawing score, the number of landmarks noticed, and the average number of landmarks used during the learning phase.

### Questionnaires

#### McGill GPS questionnaire

We developed and administered the McGill GPS questionnaire, which included questions about participants’ use of GPS. This questionnaire was adapted from the Driving Habits Questionnaire^35^ and was used to assess lifetime GPS experience, i.e. the lifetime number of hours that participants had been using a GPS, and the lifetime number of hours that they had been driving. Experimenters administering the questionnaire asked detailed questions about how often and how long participants use GPS, and whether their GPS use changed throughout the weeks, months, and years preceding the experiment, which allowed us to estimate lifetime GPS use and hours of GPS use since pre-test. For example, experimenters asked questions such as “Currently, how much time do you use GPS in a typical week?”, “How long would you say you have used GPS for this weekly amount?”, and “Has the amount of time that you use GPS changed over the years?”. The questionnaire also contains questions regarding why participants obtained a GPS and how they used it.

We developed two 5-point Likert scales relating to different aspects of GPS use: the GPS reliance scale (Supplementary Fig. S1) and the sense of GPS dependence scale (Supplementary Fig. S2). Participants are asked to consider the past month as they answer the questions. The ‘GPS reliance’ scale has seven items and evaluates the frequency at which people rely on GPS in various situations (e.g. “How often do you use a GPS to travel new routes to an unfamiliar destination?”; “How often do you use a GPS to travel new routes to a previously visited destination?”). The ‘Sense of GPS dependence’ scale has 13 items and measures the extent to which people feel dependent on their GPS (e.g., “I get lost easily in a new environment when I am not using a GPS”; “I feel anxious when driving without a GPS”). Each response corresponds to a number and the total score is calculated based on the sum of the individual responses. In the GPS reliance scale, the score for Question 5 is reversed. In the sense of GPS dependence scale, the scores for Questions 3, 6, and 12 are reversed. We conducted a pilot study to validate these scales. Fourteen healthy young drivers who drove regularly (at least 4 days a week) and regularly used GPS (at least once a week) were included in the pilot study (six women and eight men; mean age: 25.93 ± 4.32 years old). To determine the validity of the scales, we measured their internal consistency by calculating Cronbach’s alpha. The GPS reliance scale had a Cronbach’s alpha of 0.79 while the sense of GPS dependence scale had an alpha of 0.77, indicating that both scales have good internal consistency. The test-retest reliability of the scales was not assessed in this pilot study; however, we used the pre/post data in the longitudinal study to examine this question. The test-retest reliability for the 13 participants was good, as the correlations were moderate to high for the GPS reliance scale (*r* = 0.60, Bootstrap BCa 95% CI [0.21, 0.84]) and the sense of GPS dependence scale (*r* = 0.55, Bootstrap BCa 95% CI [0.14, 0.81]).

### Neuropsychological tests

To characterize our samples of participants in terms of neuropsychology, we administered the Rey Auditory Verbal Learning Task (RAVLT)^36^ to assess verbal memory, the Rey-Osterrieth Complex Figure (ROCF)^37^ to assess visuo-spatial memory, the Test of Non-verbal Intelligence-3 (TONI-3)^38^ to evaluate non-verbal intelligence, and the Santa Barbara Sense of Direction scale (SBSOD)^39^ to assess subjective sense of direction. The cross-sectional and longitudinal samples’ averages for the RAVLT delayed recall, ROCF delayed recall, TONI-3 quotient, and SBSOD are shown in Table 1.

#### Analysis

We used SPSS Statistics 20 (IBM) to conduct all analyses. We correlated our GPS measures with our navigational dependent variables using Pearson correlations. Bootstrapped bias-corrected and accelerated 95% confidence intervals (Bootstrap BCa 95% CI) were calculated to account for deviations from parametric assumptions and to determine significance. We used bootstrapping to simulate 1000 datasets by resampling from one sample dataset with replacement. Such resampling methods are beneficial because of their inherent correction for multiple comparisons^40,41^. We bootstrapped confidence intervals, which are more informative as they estimate the true value of the population. This renders them more precise and more robust than *p* values^42–44^, even more so when bootstrapping is used^40^. Resampling methods are also advantageous in that they estimate Type I and Type II error rates more accurately than classical *p* value adjustment methods^41^, since the experimental data is compared with a chance distribution. Bootstrapping is non-parametric and as such does not require transforming the data when it is not normally distributed^41,45^. We calculated one-tailed confidence intervals for all analyses pertaining to GPS variables and navigational dependent variables as they were hypothesis-driven.

To make sure that any effect of GPS experience would not be confounded by driving experience, we correlated lifetime driving experience (in hours) with our navigational variables of interest. No correlations were significant (all Bootstrap BCa 95% CI crossed 0), therefore lifetime driving experience was not used as a covariate in our analyses.

In the longitudinal study, we calculated the change in performance between pre- and post-testing by subtracting pre-testing scores from post-testing scores. We then ran partial correlations between these scores and i) the amount of GPS use (in hours) between pre- and post-testing, covarying with performance at pre-test as well as lifetime GPS experience (in hours) at pre-test as participants did not start equal; and ii) the change in GPS reliance scores, covarying with performance at pre-test and GPS reliance scores at baseline. Additionally, neuropsychological scores did not change from pre- to post-testing (all Bootstrap BCa 95% CI crossed 0), therefore we did not use any neuropsychological scores as covariates.

An association between greater GPS habits and lower spatial memory use could be confounded by subjective sense of direction, as we might expect that greater GPS habits would be associated with a lower subjective sense of direction. For example, someone who feels they have a poor sense of direction may use GPS more frequently as a way to compensate. Therefore, to infer causality, we correlated GPS habits with subjective sense of direction, as assessed with the SBSOD, using Pearson correlations. Whenever there was a significant correlation, we used SBSOD scores as a covariate in the analyses estimating the relationship between GPS habits and navigation variables. We also correlated SBSOD scores with navigational variables to examine whether subjective sense of direction is related to better objective navigational performance.

Other analyses included test-retest reliability analysis, where we correlated the scores on the GPS scales between pre and post using Pearson correlations, verifying that the longitudinal sample did not significantly differ from the remaining participants in the original sample in terms of demographics and navigational performance, using independent samples t-tests for continuous variables (e.g., education) and Chi-square test for dichotomous variables (e.g., sex).

## Results

### Cross-sectional study

We tested 50 healthy young regular drivers who drive a minimum of four days a week and characterized their GPS habits using our McGill GPS questionnaire. This questionnaire was designed to assess lifetime GPS experience, as well as GPS reliance and sense of dependence on GPS. We also evaluated participants’ subjective sense of direction using the SBSOD.

We assessed participants’ navigation abilities by administering two virtual radial arm mazes: the Concurrent Spatial Discrimination Learning Task (CSDLT)^28,29^ and the 4-on-8 Virtual Maze (4/8 VM)^32,33^, in which either the spatial memory strategy or the stimulus-response strategy can be used to solve the task. In both of these tasks, participants have to learn the location of objects within a radial arm maze that is surrounded by a rich landscape and environmental landmarks. We identify four broad categories of variables: learning (how quickly participants learn about their environment, using one strategy or the other; CSDLT and 4/8 VM number of trials to criterion and average learning errors), navigation strategy use (CSDLT first probe trial, both probe trials combined, flexibility/rigidity as measured by the second probe trial of the CSDLT, 4/8 VM navigation strategy score), cognitive mapping (4/8 VM map drawing), and landmark encoding and reliance (4/8 VM probe errors, average number of landmarks used, and number of landmarks noticed).

**Figure 2:**
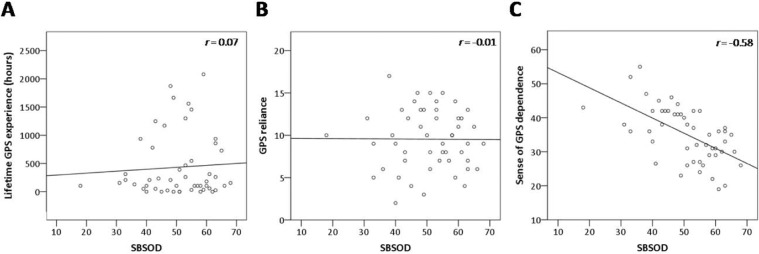
Associations between subjective sense of direction and GPS variables. Can poor spatial memory explain why certain participants used GPS to a large extent in their lifetime? If this were to be the case, we would expect to find an association between participants’ subjective sense of direction and their GPS use. There was no significant correlation between SBSOD scores and either (**A**) lifetime GPS experience, *r* = 0.07, Bootstrap BCa 95% CI [−0.16, 0.27]; or (**B**) GPS reliance, *r* = −0.01, Bootstrap BCa 95% CI [−0.24, 0.25]. These findings indicate that participants who used GPS extensively, or those who relied on GPS more in everyday situations, likely did not do so as a way to compensate for a subjectively poor sense of direction. However, there was a significant relationship between SBSOD scores and (**C**) sense of GPS dependence, *r* = −0.58, Bootstrap BCa 95% CI [−0.73, 0.−42]. Therefore, we used SBSOD scores as a covariate in the sense of GPS dependence analyses in the cross-sectional study. SBSOD: Santa Barbara Sense of Direction scale.

We determined whether SBSOD scores should be used as a covariate in our analyses. There was no significant correlation between lifetime GPS experience and SBSOD scores (*r* = 0.07, Bootstrap BCa 95% CI [−0.16, 0.27]) (Fig. 2A). Thus, it does not appear that participants who used GPS for more hours did so as a result of a subjectively poor sense of direction. With regards to the other GPS variables, there was no significant relationship between SBSOD and GPS reliance (*r* = −0.01, Bootstrap BCa 95% CI [−0.24, 0.25]) (Fig. 2B), however there was a significant relationship between SBSOD and sense of dependence on GPS (*r* = −0.58, Bootstrap BCa 95% CI [−0.73, −0.42]), whereby greater sense of dependence on GPS was associated with lower subjective sense of direction. Therefore, SBSOD scores were used as a covariate in the analyses pertaining to sense of dependence on GPS.

### Concurrent spatial discrimination learning task

**Figure 3:**
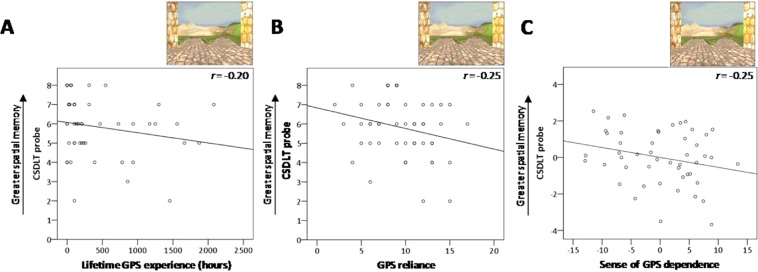
Concurrent Spatial Discrimination Learning Task results. (**A**) There is a significant negative correlation between lifetime GPS experience and performance on the first probe trial of the CSDLT, *r* = −0.22, Bootstrap BCa 95% CI [−0.41, −0.01] (here, for consistency with the other graphs, we show a marginally significant negative correlation between lifetime GPS experience and the two CSDLT probe trials, *r* = −0.20, Bootstrap BCa 95% CI [−0.41, 0.01]). There are also significant negative correlations between the two CSDLT probe trials and (**B**) scores on the GPS reliance scale, which assesses the frequency at which people use GPS in various situations, *r* = −0.25, Bootstrap BCa 95% CI [−0.44, −0.03], and (**C**) scores on the sense of GPS dependence scale, *r* = −0.25, Bootstrap BCa 95% CI [−0.34, −0.05] (covaried with SBSOD scores). Note that this graph shows plotted residuals from the partial correlation, where SBSOD scores were regressed out of the variables of interest.

We found a significant negative correlation between lifetime GPS experience (in hours) and performance on the first probe trial of the CSDLT (*r* = −0.22, Bootstrap BCa 95% CI [−0.41, −0.01]) and a marginally significant negative correlation with performance on both probe trials combined (*r* = −0.20, Bootstrap BCa 95% CI [−0.41, 0.01]) (Fig. 3A), indicating that people with more lifetime GPS experience use hippocampus-dependent spatial memory strategies to a lesser extent. Other lifetime GPS experience correlations were non-significant (i.e., number of trials to criterion, second probe trial; all Bootstrap BCa 95% CI crossed 0; see Table 2).

**Table 2:** Correlations between GPS habits and navigation performance in the cross-sectional and longitudinal studies.

	Cross-sectional study			Longitudinal study	
	Lifetime GPS experience (hours)	GPS reliance	Sense of GPS dependence	Hours of GPS use since pre-test	GPS reliance (post-pre)
**CSDLT**					
Number of trials to criterion	r = −0.06 [−0.32, 0.18]	r = 0.08 [−0.14, 0.27]	r = 0.15 [−0.09, 0.34]	r = 0.12 [−0.29, 0.65]	r = 0.62 [0.18, 0.91]*
Probe 1	r = −0.22 [−0.41, −0.01]*	r = −0.17 [−0.39, 0.07]	r = 0.01 [−0.24, 0.27]	r = 0.05 [−0.60, 0.60]	r = −0.33 [−0.81, 0.55]
Probe 2	r = −0.08 [−0.36, 0.15]	r = −0.21 [−0.45, 0.05]	r = −0.33 [−0.49, −0.14]*	r = −0.68 [−0.91, −0.10]*	r = −0.47 [−0.75, −0.28]*
Probe 1 and 2	r = −0.20 [−0.41, 0.01]	r = −0.25 [−0.44, −0.03]*	r = −0.25 [−0.43, −0.05]*	r = −0.15 [−0.72, 0.50]	r = −0.35 [−0.87, 0.58]
**4/8 VM**					
Number of trials to criterion	r = −0.12 [−0.29, 0.07]	r = 0.08 [−0.13, 0.28]	r = −0.13 [−0.36, 0.10]	r = 0.16 [−0.64, 0.92]	r = 0.17 [−0.65, 0.74]
Average learning errors on Part 2	r = 0.02 [−0.21, 0.27]	r = 0.20 [−0.03, 0.44]	r = −0.16 [−0.42, 0.11]	r = −0.18 [−0.58, 0.28]	r = 0.21 [−0.43, 0.69]
Probe errors	r = 0.04 [−0.20, 0.27]	r = 0.16 [−0.09, 0.41]	r = 0.09 [−0.16, 0.34]	r = 0.02 [−0.44, 0.69]	r = 0.42 [−0.37, 1.00]
Map drawing	r = −0.22 [−0.41, −0.02]*	r = −0.26 [−0.51, 0.06]	r = −0.13 [−0.36, 0.14]	r = −0.52 [−0.79, −0.21]*	r = −0.64 [−0.83, −0.50]*
Navigation strategy score	r = −0.20 [−0.38, −0.02]*	r = 0.04 [−0.22, 0.31]	r = −0.02 [−0.25, 0.20]	r = −0.27 [−0.77, 0.44]	r = 0.19 [−0.37, 0.70]
Average number of landmarks used	r = −0.21 [−0.38, −0.05]*	r = −0.05 [−0.27, 0.23]	r = 0.01 [−0.23, 0.24]	r = −0.32 [−0.68, −0.05]*	r = −0.01 [−0.56, 0.46]
Number of landmarks noticed	r = −0.26 [−0.42, −0.09]*	r = −0.27 [−0.49, −0.04]*	r = −0.07 [−0.32, 0.21]	r = −0.57 [−0.95, 0.61]	r = −0.67 [−0.90, −0.07]*

Correlation *r* coefficients are shown with bootstrap bias-corrected 95% confidence intervals in brackets. Sense of GPS dependence correlations were covaried with SBSOD scores. Longitudinal analyses were covaried with navigation performance at baseline and GPS measure at baseline. Significant correlations (Bootstrap BCa 95% CI not overlapping with 0) are denoted with *.

CSDLT: Concurrent Spatial Discrimination Learning Task; 4/8 VM: 4-on-8 Virtual Maze.

With regards to GPS reliance, there was a significant negative correlation with both probe trials combined (*r* = −0.25, Bootstrap BCa 95% CI [−0.44, −0.03]) (Fig. 3B), which indicates that as GPS reliance increases, spatial memory strategy use decreases. Other GPS reliance correlations were non-significant (number of trials to criterion, first and second probe trials; Table 2). In terms of sense of GPS dependence, there was a significant positive correlation between sense of GPS dependence scores and the second probe trial of the CSDLT (*r* = −0.33, Bootstrap BCa 95% CI [−0.49, −0.14], covaried with the SBSOD). This indicates that those who feel more dependent on their GPS have a lower ability to learn from their mistakes in the first probe trial, regardless of their subjective sense of direction. There was also a significant correlation with both probe trials combined (*r* = −0.25, Bootstrap BCa 95% CI [−0.43, −0.05], covaried with the SBSOD) (Fig. 3C), indicating a lower use of spatial memory strategies in those who feel more dependent on their GPS, although this effect was driven by performance on the second probe trial because the correlation with the first probe trial was near zero (*r* = 0.01, Bootstrap BCa 95% CI [−0.24, 0.27]). No other correlation (number of trials to criterion) with sense of GPS dependence scores was significant (Bootstrap BCa 95% CI crossed 0; Table 2).

We also examined the question of whether perceived spatial abilities are related to actual navigational performance on the CSDLT. SBSOD scores were significantly related to faster learning (*r* = −0.26, Bootstrap BCa 95% CI [−0.48, −0.03]) and, surprisingly, to lower performance on the second probe trial (*r* = −0.28, Bootstrap BCa 95% CI [−0.45, −0.09]) and on both trials combined (*r* = −0.25, Bootstrap BCa 95% CI [−0.45, −0.02], although this was mostly driven by performance on the second probe trial as the correlation with the first probe trial was non-significant: *r* = −0.12, Bootstrap BCa 95% CI [−0.38, 0.14]).

### 4-on-8 virtual maze (4/8 VM)

**Figure 4:**
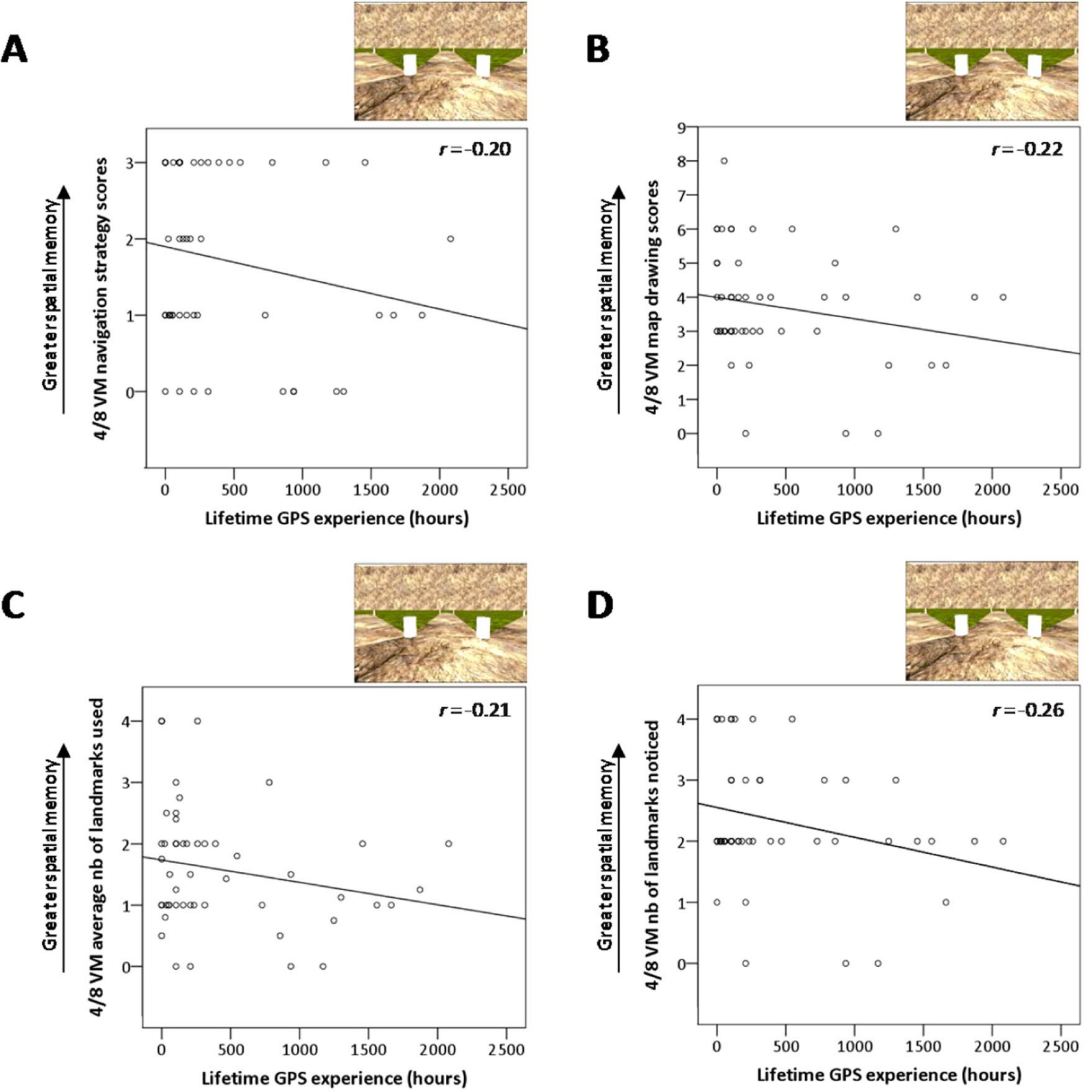
4-on-8 virtual maze results. (**A**) We found a significant negative correlation between lifetime GPS experience and navigation strategy scores, *r* = −0.20, Bootstrap BCa 95% CI [−0.38, −0.02]. (**B**) There is a significant negative correlation between lifetime GPS experience and map drawing scores, *r* = −0.22, Bootstrap BCa 95% CI [−0.41, −0.02], indicating that greater GPS experience is associated with a lesser ability to form cognitive maps. (**C**) There is a significant negative correlation between lifetime GPS experience and both the average number of landmarks used during the learning phase of the 4/8 VM, *r* = −0.21, Bootstrap BCa 95% CI [−0.38, −0.05] as well as (**D**) the number of landmarks noticed in the environment, *r* = −0.26, Bootstrap BCa 95% CI [−0.42, −0.09], indicating that those with greater GPS experience encoded less landmark information.

We found a significant negative correlation between lifetime GPS experience and navigation strategy scores (*r* = −0.20, Bootstrap BCa 95% CI [−0.38, −0.02]) (Fig. 4A). This indicates that individuals with more lifetime GPS experience use hippocampus-dependent spatial memory strategies to a lesser extent, concordant with the CSDLT probe results. People with greater lifetime GPS experience also had more difficulty forming a cognitive map, as evidenced by a significant negative correlation between lifetime GPS experience and map drawing scores (*r* = −0.22, Bootstrap BCa 95% CI [−0.41, −0.02]) (Fig. 4B). This can at least in part be explained by the fact that people with more lifetime GPS experience encoded fewer landmarks, as there were significant negative correlations between lifetime GPS experience and the average number of landmarks used while solving the task (*r* = −0.21, Bootstrap BCa 95% CI [−0.38, −0.05]) (Fig. 4C), as well as the number of landmarks they noticed in the environment (*r* = −0.26, Bootstrap BCa 95% CI [−0.42, −0.09]) (Fig. 4D). All other lifetime GPS experience correlations were non-significant (number of trials to criterion, average learning errors, probe errors; all Bootstrap BCa 95% CI crossed 0; Table 2).

Does worse spatial memory in those who use GPS more extensively solely reflect their decreased tendency to use spatial memory strategies in favor of stimulus-response strategies, or does it also reflect their poor abilities when they do use spatial strategies? To investigate this, we examined associations in only those who used spatial memory strategies during task acquisition (n = 17). Greater lifetime GPS experience was significantly associated with a lower number of landmarks noticed only (*r* = −0.37, Bootstrap BCa 95% CI [−0.63, −0.04]). For all other measures, the associations were non-significant but did reflect the hypothesized direction (number of trials to criterion: *r* = 0.20, Bootstrap BCa 95% CI [−0.20, 0.58]; average learning errors: *r* = 0.40, Bootstrap BCa 95% CI [−0.14, 0.69]; map drawing: *r* = −0.15, Bootstrap BCa 95% CI [−0.41, 0.05]; average number of landmarks used: *r* = −0.19, Bootstrap BCa 95% CI [−0.47, 0.12]). Non-significant effects may be due to the small sample size (n = 17). Importantly, all these correlations indicate that, within spatial memory strategy users, lifetime GPS experience is associated with worse spatial memory abilities. Therefore, GPS use not only affects the extent to which people use spatial memory strategies, but also their ability to use spatial memory abilities effectively.

With regards to GPS reliance, there was a significant correlation between GPS reliance and the number of landmarks noticed (*r* = −0.27, Bootstrap BCa 95% CI [−0.49, −0.04]), indicating that, as reliance on GPS increased, people noticed fewer landmarks in the environment. All other correlations with GPS reliance (number of trials to criterion, average number of learning errors, probe errors, map drawing, navigation strategy, and number of landmarks used) were non-significant (Bootstrap BCa 95% CI crossed 0; Table 2). None of the sense of GPS dependence correlations with 4/8 VM variables (with SBSOD as a covariate) were significant (BCa 95% CI crossed 0; Table 2).

We investigated whether perceived sense of direction was related to navigational performance in the 4/8 VM. There were no significant correlations between SBSOD scores and 4/8 VM variables (all Bootstrap BCa 95% CI crossed 0).

### Longitudinal study

GPS reliance scores increased from pre- to post-testing (post vs. pre: mean difference = 5.50 [7.74, 2.96]), however sense of GPS dependence scores did not (Bootstrap BCa 95% CI crossed 0). We therefore used GPS use since pre-testing (‘Hours of GPS use since pre-test’), post- minus pre-GPS reliance scores (‘Post-pre GPS reliance scores’), and post- minus pre-SBSOD scores as independent variables and post- minus pre-performance scores on the navigation tasks as dependent variables. We hypothesized that those who used GPS more extensively between pre- and post-testing would exhibit a steeper decline in spatial memory and cognitive mapping.

**Figure 5:**
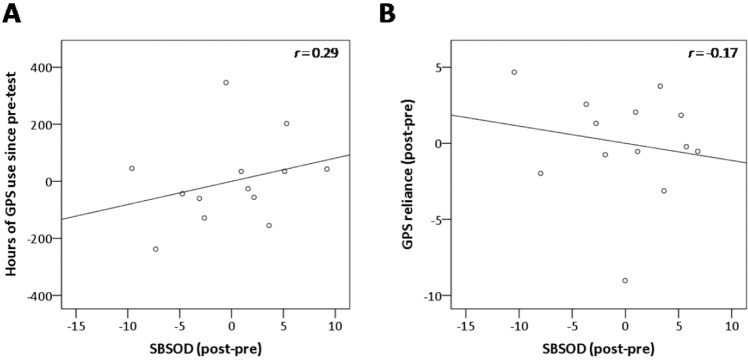
There is no association between a decline in subjective sense of direction and GPS use or reliance since pre-test. Could naturally-occurring spatial memory decline explain greater GPS use since pre-test? If this were to be the case, we would expect a decline in subjective sense of direction to be associated with greater change in GPS use and reliance. There was no significant correlation between change in SBSOD scores and either (**A**) hours of GPS use since pre-test, *r* = 0.29, Bootstrap BCa 95% CI [−0.92, 0.98] (covaried with SBSOD at baseline and lifetime GPS experience at baseline); or (**B**) GPS reliance, *r* = −0.17, Bootstrap BCa 95% CI [−0.72, 0.58] (covaried with SBSOD at baseline and GPS reliance at baseline). Note that the graphs show plotted residuals from the partial correlations, where covariates were regressed out of the variables of interest. Spatial memory decline was observed without a concurrent decline in subjective sense of direction. Thus, spatial memory decline likely occurred as a result of GPS use, rather than the other way around. SBSOD: Santa Barbara Sense of Direction scale.

If a decline in spatial memory prompted participants to use GPS more, or to become more reliant on GPS, then we would expect that hours of GPS use since pre-test/change in GPS reliance would be associated with a decline in subjective sense of direction. Thus, to eliminate subjective sense of direction as a potential confounding factor, we determined whether change in SBSOD scores should be used as a covariate in the analyses probing GPS habits and navigation performance over time. There was no significant correlation between the post- minus pre-SBSOD and either GPS use since pre-testing scores (*r* = 0.29, Bootstrap BCa 95% CI [−0.92, 0.98], covaried with SBSOD and lifetime GPS experience at baseline) (Fig. 5A), or post- minus pre-GPS reliance scores (*r* = −0.17, Bootstrap BCa 95% CI [−0.72, 0.58], covaried with SBSOD and GPS reliance at baseline) (Fig. 5B). Thus, using GPS more extensively or becoming more reliant on it is not associated with a decline in subjective sense of direction over time. Any decline in spatial memory can therefore be attributed to a greater use of/reliance on GPS. As such, change in SBSOD scores was not used as a covariate in the longitudinal analyses.

### Concurrent spatial discrimination learning task (CSDLT)

**Figure 6:**
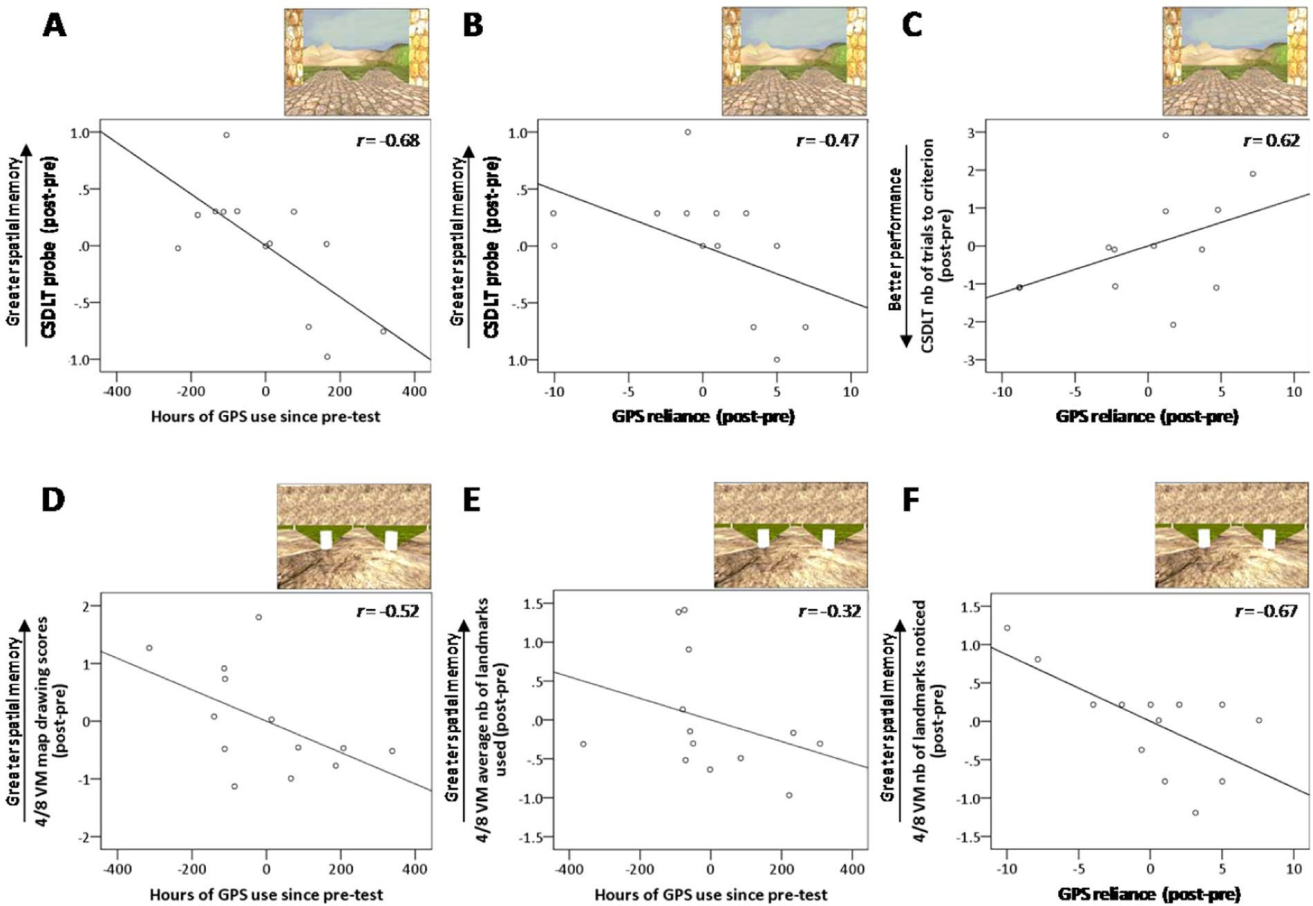
Longitudinal results show decreased spatial memory as a function of increased GPS use and reliance with time. Thirteen participants were retested three years after initial testing. We investigated the change in navigational variables between pre and post-testing, covarying with performance and lifetime GPS experience at baseline. Note that the graphs show plotted residuals from the partial correlations, where covariates were regressed out of the variables of interest. For example, although the ‘Hours of GPS use since pre-test’ variable takes on negative values in the graphs, all original (unregressed) datapoints are in the positive range because all participants used GPS over the three-year period. (**A**) In the CSDLT, there is a significant negative correlation between hours of GPS use since pre-test and post-pre performance on the second probe trial, *r* = −0.68, Bootstrap BCa 95% CI [−0.91, −0.10]. (**B**) There is also a significant negative correlation between post-pre GPS reliance scores and post-pre performance on the second CSDLT probe trial, *r* = −0.47, Bootstrap BCa 95% CI [−0.75, −0.28]. (**C**) There is a significant negative correlation between post-pre GPS reliance scores and the number of trials required to reach the learning criterion of the CSDLT, *r* = 0.62, Bootstrap BCa 95% CI [0.18, 0.91], suggesting that more frequent reliance on GPS in various situations leads to greater difficulty in learning the location of objects. (**D**) On the 4/8 VM, there is a significant negative correlation between hours of GPS use since pre-test and post-pre map drawing scores, *r* = −0.52, Bootstrap BCa 95% CI [−0.79, −0.21], indicating that GPS use leads to a lower ability to form cognitive maps. (**E**) There is a significant negative correlation between hours of GPS use since pre-test and post-pre average number of landmarks used, *r* = −0.32, Bootstrap BCa 95% CI [−0.68, −0.05] as well as (**F**) a significant negative correlation between post-pre GPS reliance scores and the post-pre number of landmarks noticed in the environment, *r* = −0.67, Bootstrap BCa 95% CI [−0.90, −0.07], indicating that increased GPS use leads to decreased landmark encoding.

In the CSDLT, there was a significant negative correlation between post-pre performance on the second probe trial and both hours of GPS use since pre-test (*r* = −0.68, Bootstrap BCa 95% CI [−0.91, −0.10], covaried with lifetime GPS experience and probe 2 score at baseline) (Fig. 6A) and post-pre GPS reliance scores (*r* = −0.47, Bootstrap BCa 95% CI [−0.75, −0.28], covaried with GPS reliance and probe 2 score at baseline) (Fig. 6B), suggesting that using GPS leads to a decreased use of spatial memory strategies. There was also a significant negative correlation between post-pre GPS reliance scores and the number of trials required to reach the learning criterion of the CSDLT (*r* = 0.62, Bootstrap BCa 95% CI [0.18, 0.91], covaried with GPS reliance and number of trials to criterion at baseline) (Fig. 6C), indicating that more frequent GPS use resulted in greater difficulty in learning the location of the objects. All other correlations were non-significant (Bootstrap BCa 95% CI crossed 0; Table 2).

### 4-on-8 virtual maze (4/8 VM)

On the 4/8 VM, there was a significant negative correlation between hours of GPS use since pre-test and post-pre map drawing scores (*r* = −0.52, Bootstrap BCa 95% CI [−0.79, −0.21], covaried with lifetime GPS experience and map drawing at baseline) (Fig. 6D). Post-pre GPS reliance scores also exhibited a significant negative correlation with post-pre map drawing scores (*r* = −0.64, Bootstrap BCa 95% CI [−0.83, −0.50], covaried with GPS reliance and map drawing at baseline). Thus, those who used GPS more extensively since the initial visit exhibited a steeper decline in their cognitive mapping abilities. They also exhibited a steeper decline in landmark encoding, as evidenced by a significant negative correlation between hours of GPS use since pre-test and post-pre average number of landmarks used (*r* = −0.32, Bootstrap BCa 95% CI [−0.68, −0.05], covaried with lifetime GPS experience and number of landmarks used at baseline) (Fig. 6E), and a significant negative correlation between post-pre GPS reliance scores and the post-pre number of landmarks noticed in the environment (*r* = −0.67, Bootstrap BCa 95% CI [−0.90, −0.07], covaried with GPS reliance and number of landmarks noticed at baseline) (Fig. 6F). All other correlations were non-significant (BCa 95% CI crossed 0; Table 2).

## Discussion

Using GPS to navigate from one point to another removes the need to pay attention to our surroundings and to internally update our position as we travel. We investigated the navigational abilities and characteristics of people with different levels of GPS experience, reliance on GPS in various situations, and sense of dependence on GPS. We hypothesized that people with greater GPS habits would use spatial memory strategies to a lesser extent and that they would show poorer spatial memory when they are required to find their way in an environment that they have experienced without GPS. Both the cross-sectional and longitudinal studies support this hypothesis (Figs. 3, 4, 6), and the longitudinal study further supports a causal relationship between GPS habits and poor spatial memory—that is, using GPS leads to a decline in spatial memory. While the reverse is possible, i.e. that declining spatial memory would lead to increased use of GPS, it is unlikely as there was no association between GPS use or reliance and a subjectively poorer sense of direction (Figs. 2, 5). While there was a relationship between a lower sense of GPS dependence and lower subjective sense of direction (Fig. 2), further analysis shows that there are nonetheless effects of sense of GPS dependence on spatial memory that are robust, even while accounting for subjective sense of direction. Moreover, we show that the relationship between GPS use and spatial memory decline is dose-dependent, in that those who used GPS to a greater extent between the two time points demonstrated a larger decline in spatial memory. This effect was observed in various facets of spatial memory, including the extent of use of spatial memory strategies, cognitive mapping, landmark encoding, and learning. Poorer spatial memory in those who use GPS extensively is not solely due to their greater tendency to use stimulus-response strategies at the expense of spatial memory strategies, as these associations exist even within spatial memory strategy users. Our main findings are listed in Table 2, which makes it clear that each of our GPS measures, namely GPS use (lifetime GPS experience and hours of GPS use since pre-test), GPS reliance, and sense of dependence on GPS, are related to several facets of spatial memory. Although these GPS measures represent different constructs, each of their effects on spatial memory are not limited to any single facet and there is substantial overlap in the navigational variables they act on. This is reflected in the significantly positive correlations between GPS variables. Thus, we will not seek to disambiguate the unique effects displayed by each GPS construct as we do not believe it is possible to do so in the current study. Instead, we aggregate the results for the GPS variables together under the umbrella of GPS habits and discuss our findings below, organizing them by facets of spatial memory (strategy use, cognitive mapping, landmark encoding and reliance, and learning.

First, we found negative correlations between GPS habits and the extent of spatial memory strategy use, i.e. the greater the GPS habits, the lower the reliance on spatial memory strategies. In the CSDLT, there were longitudinal and cross-sectional effects of lifetime GPS experience, hours of GPS use since pre-test, and GPS reliance on spatial memory strategy use and flexibility to learn from one’s mistakes. There was also an effect of sense of GPS dependence on spatial memory strategy use and flexibility in the cross-sectional study. In the 4/8 VM, there was a cross-sectional effect of lifetime GPS experience on spatial memory strategy use. The longitudinal effect was non-significant, however the direction of the correlation was consistent with the cross-sectional effect (*r* = −0.27, Bootstrap BCa 95% CI [−0.77, 0.44]; Table 2). It is possible that a significant effect would emerge with increased power, but this would have to be confirmed in a larger study. These findings indicate that extended GPS use is associated with reduced use of spatial memory strategies and increased use of stimulus-response strategies. This is consistent with the idea that navigating with GPS is similar to using a stimulus-response strategy, however it is also possible that navigating with GPS simply acts on the same brain system as using stimulus-response strategies.

GPS habits were also associated with lower cognitive mapping abilities, as there were longitudinal effects of hours of GPS use since pre-test and GPS reliance and cross-sectional effects of lifetime GPS experience on map drawing scores in the 4/8 VM. These results suggest that using GPS renders individuals less able to form an accurate mental representation of their surroundings when they are navigating without GPS.

A decrease in cognitive mapping abilities may at least in part be explained by reduced landmark encoding and reliance. There were longitudinal and cross-sectional effects of hours of GPS use since pre-test and lifetime GPS experience on the average number of landmarks used, as well as a cross-sectional and longitudinal effect of GPS reliance and a cross-sectional effect of lifetime GPS experience on the number of landmarks that were noticed. Thus, using GPS is associated with noticing and using fewer landmarks, possibly because individuals are less required to pay attention to their surroundings when they consistently drive with GPS.

Finally, there is some evidence that learning the location of the rewarded paths in the CSDLT was more difficult with increasing GPS habits, as there was a longitudinal effect of GPS reliance on the number of trials to criterion. Thus, using GPS is associated with reduced navigational learning, although there were no effects of GPS use on learning in the 4/8 VM. The lack of an effect in the 4/8 VM could be explained by the fact that one does not have to use any landmarks on the 4/8 VM, for example when using stimulus-response strategies that only depend on the constant start position as the stimulus that elicits a response on the maze, while landmark use in the CSDLT is necessary, even when using stimulus-response strategies where the stimulus that elicits a response is a landmark in the environment (e.g., “When I see the pyramids, go left”).

Did spatial memory decline lead to increased GPS use, or did increased GPS use lead to spatial memory decline? If participants felt that their sense of direction was worse and used GPS more extensively as a way to compensate for their diminished abilities, then there should be an association between subjective sense of direction and amount of GPS use. However, the cross-sectional study showed no association between low subjective sense of direction and higher lifetime GPS experience, and the longitudinal study similarly did not show an association between low subjective sense of direction and greater hours of GPS use over the three-year period, despite the decline in spatial memory. In other words, those who used GPS more did not do so because they felt that they had a poor sense of direction. For this reason, it is unlikely that participants with worse spatial memory compensated for their decreased spatial abilities by using GPS more extensively. Similarly, there was no association between subjective sense of direction and GPS reliance. We did find higher sense of GPS dependence to be related to lower subjective sense of direction, however the effects we reported were covaried with the SBSOD and are therefore not explained by subjective sense of direction. Our results suggest that more extensive GPS use led to worse decline in spatial memory, rather than the other way around. There was no association between participants’ subjective sense of direction and their performance on the 4/8 VM. This is likely because all participants performed relatively well on the task and had high functional levels of navigational ability. However, there was an association between subjective sense of direction and performance on the CSDLT, whereby those with high SBSOD scores learned more quickly on the CSDLT but performed more errors on the second probe trial. Thus, it may be that people who feel they have a good sense of direction have a false sense of confidence regarding the navigational information they have learned, and thus display more rigid behavior in the CSDLT. Regardless, subjective sense of direction did not explain the effects that GPS habits exerted on spatial memory. Additionally, our cohort was comprised of healthy young adults (mean age in cross-sectional sample: 27.6 ± 4.5; mean age in longitudinal sample at pre-test: 28.5 ± 3.9), therefore we would not expect that they would experience an age-related decline in cognitive abilities. In fact, there was no change in neuropsychological scores between pre- and post-testing. Because the follow-up session was unplanned in the initial study, few participants were able to come back three years later. Nonetheless, the effect of GPS use on spatial memory over time was quite important, yielding moderate to large effect sizes, despite accounting for subjective sense of direction. Thus, while we caution against any strong conclusions as spurious correlations are possible, these findings do suggest that GPS use may cause a decline in spatial memory with habitual and consistent use.

Our results concord with previous studies in the literature^1–4^. In Ishikawa *et al*.’s study^1^, the authors had three groups of participants walk along six routes: one group used GPS to navigate, one group used maps, and one group walked the routes without any aid after walking them once while being guided by an experimenter. Once participants reached the target destination, they had to estimate the direction of the starting point and draw the shape of the route. The authors found that the group that used GPS during navigation had poorer direction estimation and route drawing than the group of participants that had previously navigated with an experimenter. Similar results were found in studies comparing spatial information learned during GPS- or GPS-like-guided navigation and spatial information learned using traditional maps or self-guided navigation^2–5^. The fact that participants learn less accurate spatial information about GPS-guided routes supports the notion that using GPS lessens the need to pay attention to our surroundings and to internally update our position. Gardony *et al*.^5^ provide evidence that navigational aids impair spatial memory by dividing attention. However, in their study, the navigational aid did not completely disengage users; participants were informed about their proximity and bearing in relation to the target location but did not receive turn-by-turn instructions, requiring participants to make navigational decisions about their routes. Thus, we propose that it is mainly through disengagement from our environment that GPS exerts negative effects on spatial memory. Crucially, our study shows that this impairment is transferred to navigation without GPS—that is, spatial memory deficits are present when people navigate without GPS and have to rely on their own internal sense of orientation when they experience a new environment. This indicates that, over time, GPS use reduces people’s propensity to gain and memorize spatial information as well as their ability to form accurate cognitive maps.

Spatial memory critically relies on the hippocampus^6,8–11^ and people who use these strategies have greater fMRI BOLD activity and greater grey matter in the hippocampus^27,29,30,32,33,46,47^. Our findings suggest that people with greater GPS habits may rely less on their hippocampus for navigation, as they exhibit a reduced use of spatial memory strategies, reduced cognitive mapping abilities, reduced landmark encoding, and as they have more difficulty learning navigational information. This is consistent with a recent study by Spiers’ group^48^, in which participants who were given instructions on where to turn at decision points while navigating in a film simulation, akin to using GPS, exhibited less fMRI BOLD activity in the hippocampus than when participants self-guided and had to make decisions unaided. This study also showed that hippocampal activity was correlated with navigational demands in the self-guided condition but not the GPS-like condition. These findings indicate that using our sense of orientation and actively taking part in navigation allows us to maintain our spatial memory abilities and engage the hippocampus. On the other hand, not doing so may have adverse effects on spatial memory, as we have shown here, and may negatively impact the integrity of the hippocampus. It is also possible that the over-engagement of the stimulus-response learning system, through the use of GPS, exerts negative effects on spatial memory due to the competitive nature of the two systems. Several studies have shown, for example, that lesioning the hippocampus or other structures that are part of the neural circuit mediating spatial memory results in impaired spatial memory but facilitates stimulus-response learning^15,18,25,49^. Repeatedly and habitually tapping into the stimulus-response learning system may likewise hinder spatial memory. Further research should seek to discern the neural circuitry that is engaged during GPS-guided navigation, the potential effects of long-term GPS use on the hippocampus, and should seek to replicate our findings in a larger sample.

In summary, our findings suggest that regularly using GPS affects spatial memory in a dose-dependent manner—that is, the greater use of GPS, the greater decline in spatial memory over time. The lack of a concurrent decline in subjective sense of direction suggests that this relationship is likely to be causal. Studies in the literature that compared GPS use to other navigation aids, such as maps, suggest that the amount of engagement is an important factor for spatial learning. Indeed, using a GPS renders one less engaged in navigation and less cognizant of landmarks compared to reading a map or navigating without an aid. This is especially relevant in communities where wayfinding plays an important role, such as among the Inuit. The Inuit traditionally rely heavily on wind currents, snowdrift patterns, and astronomical information, amongst other things, for navigation^50^. In the harsh environmental conditions of some northern Canadian regions, then, the most direct route between two points as determined by GPS is rarely the most optimal, as it does not account for the safety of the route. Too heavy of a reliance on GPS can be life-threatening in situations where the technology breaks down^50^. Future GPS technologies may opt to include landmarks in GPS-guided instructions as a way to reengage users with their surroundings. As Aporta and Higgs^50^ have noted, “there is a sense of fulfillment and accomplishment in being able to relate fully to the activity we perform and to the environment in which we are.”

## Supplementary information

Supplementary Information.

## Data Availability

The data that support the findings of this study are available from the corresponding author upon reasonable request.

## References

[1] Ishikawa T, Fujiwara H, Imai O, Okabe A (2008). Wayfinding with a GPS-based mobile navigation system: A comparison with maps and direct experience. J. Environ. Psychol..

[2] Burnett, G. E. & Lee, K. In *International conference of traffic and transport psychology* (2005).

[3] Wessel, G., Ziemkiewicz, C., Chang, R. & Sauda, E. In *Proceedings of the International Conference on Advanced Visual Interfaces*. 207–214 (ACM) (2010).

[4] Münzer S, Zimmer HD, Baus J (2012). Navigation assistance: A trade-off between wayfinding support and configural learning support. J. Exp. psychology: Appl..

[5] Gardony AL, Brunyé TT, Mahoney CR, Taylor HA (2013). How navigational aids impair spatial memory: Evidence for divided attention. Spat. Cognition Computation.

[6] O’Keefe, J. & Nadel, L. *The hippocampus as a cognitive map*. (Clarendon, 1978).

[7] Tolman EC (1948). Cognitive maps in rats and men. Psychological Rev..

[8] Jarrard LE (1993). On the role of the hippocampus in learning and memory in the rat. Behav. neural Biol..

[9] Morris R, Garrud P, Rawlins J, O’Keefe J (1982). Place navigation impaired in rats with hippocampal lesions. Nature.

[10] Olton DS, Paras BC (1979). Spatial memory and hippocampal function. Neuropsychologia.

[11] Bohbot VD, Iaria G, Petrides M (2004). Hippocampal function and spatial memory: evidence from functional neuroimaging in healthy participants and performance of patients with medial temporal lobe resections. Neuropsychology.

[12] Scoville WB, Milner B (1957). Loss of recent memory after bilateral hippocampal lesions. J. Neurol. Neurosurg. Psychiatry.

[13] Eichenbaum H (1996). Is the rodent hippocampus just for ‘place’?. Curr. Opin. Neurobiol..

[14] McDonald RJ, White NM (1993). A triple dissociation of memory systems: hippocampus, amygdala, and dorsal striatum. Behav. Neurosci..

[15] Packard MG, Hirsh R, White NM (1989). Differential effects of fornix and caudate nucleus lesions on two radial maze tasks: evidence for multiple memory systems. J. Neurosci..

[16] Mishkin, M. & Petri, H. L. In *Ne*ur*ops*ychol*og*y of *memory* (eds. L. R. Squire & N. Butters) 287–296 (Guilford Press, 1984).

[17] Squire LR, Zola SM (1996). Structure and function of declarative and nondeclarative memory systems. Proc. Natl Acad. Sci. U S Am..

[18] Dahmani L (2018). An intrinsic association between olfactory identification and spatial memory in humans. Nat. Commun..

[19] Packard MG, McGaugh JL (1992). Double dissociation of fornix and caudate nucleus lesions on acquisition of two water maze tasks: further evidence for multiple memory systems. Behav. Neurosci..

[20] Packard MG, McGaugh JL (1996). Inactivation of hippocampus or caudate nucleus with lidocaine differentially affects expression of place and response learning. Neurobiol. Learn. Mem..

[21] Almey A (2014). Medial prefrontal cortical estradiol rapidly alters memory system bias in female rats: ultrastructural analysis reveals membrane-associated estrogen receptors as potential mediators. Endocrinology.

[22] de Bruin JPC, Moita MP, de Brabander HM, Joosten RN (2001). Place and response learning of rats in a Morris water maze: differential effects of fimbria fornix and medial prefrontal cortex lesions. Neurobiol. Learn. Mem..

[23] de Bruin JPC, Swinkels WAM, de Brabander JM (1997). Response learning of rats in a Morris water maze: Involvement of the medial prefrontal cortex. Behavioural brain Res..

[24] Delatour B (2000). t. & Gisquet-Verrier, P. Functional role of rat prelimbic-infralimbic cortices in spatial memory: evidence for their involvement in attention and behavioural flexibility. Behavioural brain Res..

[25] Chang Q, Gold PE (2003). Intra-hippocampal lidocaine injections impair acquisition of a place task and facilitate acquisition of a response task in rats. Behav. Brain Res..

[26] Marighetto A (1999). Knowing which and knowing what: a potential mouse model for age‐related human declarative memory decline. Eur. J. Neurosci..

[27] Dahmani L, Bohbot VD (2015). Dissociable contributions of the prefrontal cortex to hippocampus- and caudate nucleus-dependent virtual navigation strategies. Neurobiol. Learn. Mem..

[28] Etchamendy N, Konishi K, Pike GB, Marighetto A, Bohbot VD (2012). Evidence for a virtual human analog of a rodent relational memory task: A study of aging and fMRI in young adults. Hippocampus.

[29] Konishi K (2013). Decreased functional magnetic resonance imaging activity in the hippocampus in favor of the caudate nucleus in older adults tested in a virtual navigation task. Hippocampus.

[30] Konishi K, Bohbot VD (2013). Spatial navigational strategies correlate with gray matter in the hippocampus of healthy older adults tested in a virtual maze. Front. Aging Neurosci..

[31] Olton DS, Samuelson RJ (1976). Remembrance of places passed: Spatial memory in rats. J. Exp. Psychology: Anim. Behav. Process..

[32] Bohbot VD, Lerch J, Thorndycraft B, Iaria G, Zijdenbos AP (2007). Gray matter differences correlate with spontaneous strategies in a human virtual navigation task. J. Neurosci..

[33] Iaria G, Petrides M, Dagher A, Pike B, Bohbot VD (2003). Cognitive strategies dependent on the hippocampus and caudate nucleus in human navigation: variability and change with practice. J. Neurosci..

[34] Schwabe L, Bohbot VD, Wolf OT (2012). Prenatal stress changes learning strategies in adulthood. Hippocampus.

[35] Owsley C, Stalvey B, Wells J, Sloane ME (1999). Older drivers and cataract: driving habits and crash risk. J. Gerontology Ser. A: Biol. Sci. Med. Sci..

[36] Rey, A. L’examen psychologique dans les cas d’encéphalopathie traumatique. *Archives de psychologie* (1941).

[37] Meyers, J. E. & Meyers, K. R. *Rey Complex Figure Test and Recognition Trial: Professional Manual*. (Psychological Assessment Resources, 1995).

[38] Brown, L., Sherbenou, R. J. & Johnsen, S. K. *Test of Nonverbal Intelligence—Third Edition*. (Pro-Ed, 1997).

[39] Hegarty M, Richardson AE, Montello DR, Lovelace K, Subbiah I (2002). Development of a self-report measure of environmental spatial ability. Intelligence.

[40] Westfall, P. H. & Young, S. S. *Resampling-based multiple testing: Examples and methods for p-value adjustment*. Vol. 279 (John Wiley & Sons, 1993).

[41] Field, A., Miles, J. & Field, Z. (Sage, Thousand Oaks, 2012).

[42] Greenland S (2016). Statistical tests, P values, confidence intervals, and power: a guide to misinterpretations. Eur. J. Epidemiol..

[43] Poole C (2001). Low P-values or narrow confidence intervals: which are more durable?. Epidemiology.

[44] Rothman, K. J. (Mass Medical Soc, 1978).

[45] Haukoos JS, Lewis RJ (2005). Advanced statistics: bootstrapping confidence intervals for statistics with “difficult” distributions. Academic Emerg. Med..

[46] Maguire EA (2000). Navigation-related structural change in the hippocampi of taxi drivers. Proc. Natl Acad. Sci..

[47] Maguire EA, Woollett K, Spiers HJ (2006). London taxi drivers and bus drivers: a structural MRI and neuropsychological analysis. Hippocampus.

[48] Javadi A-H (2017). Hippocampal and prefrontal processing of network topology to simulate the future. Nat. Commun..

[49] McDonald RJ, Jones J, Richards B, Hong NS (2006). A double dissociation of dorsal and ventral hippocampal function on a learning and memory task mediated by the dorso‐lateral striatum. Eur. J. Neurosci..

[50] Aporta C (2005). Satellite culture: global positioning systems, Inuit wayfinding, and the need for a new account of technology. Curr. anthropology.

