# Supplementary material for: Habitual use of GPS negatively impacts spatial memory during self-guided navigation
Source: Sci Rep. 2020 Apr 14;10:6310. doi: 10.1038/s41598-020-62877-0 (PMC7156656; doi:10.1038/s41598-020-62877-0)
Supplement: Supplementary file 1 — Supplementary Information. [file 41598_2020_62877_MOESM1_ESM.pdf]

Supplemental Materials

Habitual use of GPS negatively impacts spatial memory during self-guided navigation

Louisa Dahmani[1] and Véronique D. Bohbot[2]

[1]Integrated Program in Neuroscience, McGill University, Montreal, Quebec, Canada, H3A 2B4.

[2]Douglas Mental Health University Institute, Department of Psychiatry, McGill University, Montreal, Quebec, Canada, H4H 1R3.

In the following questions, please rate the frequency for each action. Consider the past month as you answer the questions.

	Never	Sometimes	Often	Very often	Always
1. How often do you use a GPS when you are driving?	☐	☐	☐	☐	☐
2. How often do you use a GPS to travel **new** routes to an **unfamiliar** destination? For example, you are meeting friends at a new restaurant, and you are traveling there for the first time. How often do you use a GPS in such a situation?	☐	☐	☐	☐	☐
3. How often do you use a GPS to travel **new** routes to a **previously visited** destination? For example, you usually leave from home to go to a doctor's appointment. This time, however, your appointment is scheduled right after work. Therefore, you have to travel a new route to get to a destination you have visited before. How often do you use a GPS in such a situation?	☐	☐	☐	☐	☐
4. How often do you use a GPS to travel **familiar** routes to a **previously visited** destination? For example, you usually leave from home to visit your family. You are taking the same route as you always do. How often do you use a GPS in such a situation?	☐	☐	☐	☐	☐
5. How often do you try to find a **new** route, without a GPS, to a **previously visited** destination? For example, you usually travel a specific route to go to your friend's house. This time, you think you may get there faster by taking a different route. How often do you take new routes to travel to places you have visited before?	☐	☐	☐	☐	☐
6. How often do you use a GPS **to travel to** a destination outside of your current hometown, for example if you are driving from Montreal to Toronto?	☐	☐	☐	☐	☐
7. When **finding your way around a city** outside of your hometown, how often do you use a GPS? For example, you are in New York. How often do you use a GPS to explore the city?	☐	☐	☐	☐	☐

Supplementary Figure S1: GPS reliance scale of the McGill GPS questionnaire. Readers are free to use this scale, in which case the current article should be cited.

Please answer the following questions by circling 1, 2, 3, 4, or 5. Consider the past month as you answer the questions.

	Strongly disagree	1	2	3	4	5	Strongly agree
1. I feel anxious when driving without a GPS.		1	2	3	4	5	
2. I get lost easily in a new environment when I am not using a GPS.		1	2	3	4	5	
3. I can easily remember a new route after I have traveled it once using a GPS.		1	2	3	4	5	
4. My sense of direction has gotten worse since I have been using a GPS.		1	2	3	4	5	
5. I do not pay attention to landmarks when I am using a GPS.		1	2	3	4	5	
6. I feel comfortable driving to unfamiliar destinations without a GPS.		1	2	3	4	5	
7. I would rather use a GPS than read a map.		1	2	3	4	5	
8. After driving to a new destination using my GPS, I have difficulty remembering what I saw on the way.		1	2	3	4	5	
9. After reaching a destination using my GPS, I have difficulty finding my way back without using it again.		1	2	3	4	5	
10. I can only find my way using a GPS.		1	2	3	4	5	
11. While using a GPS, I am unaware of my location in the city while I am driving until I reach my destination.		1	2	3	4	5	
12. I feel comfortable driving in familiar environments without a GPS.		1	2	3	4	5	
13. I use my GPS to find alternative routes to a known destination (e.g., when there is a road block/construction).		1	2	3	4	5	

Supplementary Figure S2: Sense of GPS dependence scale of the McGill GPS questionnaire. Readers are free to use this scale, in which case the current article should be cited.
